# Tissue-Engineered Carotid Artery Interposition Grafts Demonstrate High Primary Patency and Promote Vascular Tissue Regeneration in the Ovine Model

**DOI:** 10.3390/polym13162637

**Published:** 2021-08-08

**Authors:** Larisa V. Antonova, Evgenia O. Krivkina, Viktoriia V. Sevostianova, Andrey V. Mironov, Maria A. Rezvova, Amin R. Shabaev, Vadim O. Tkachenko, Sergey S. Krutitskiy, Mariam Yu. Khanova, Tatiana Yu. Sergeeva, Vera G. Matveeva, Tatiana V. Glushkova, Anton G. Kutikhin, Rinat A. Mukhamadiyarov, Nadezhda S. Deeva, Tatiana N. Akentieva, Maxim Yu. Sinitsky, Elena A. Velikanova, Leonid S. Barbarash

**Affiliations:** 1 Research Institute for Complex Issues of Cardiovascular Diseases, 650002 Kemerovo, Russia; antonova.la@mail.ru (L.V.A.); leonora92@mail.ru (E.O.K.); a.mir.80@mail.ru (A.V.M.); rezvovamaria@mail.ru (M.A.R.); shabar@kemcardio.ru (A.R.S.); kss911@mail.ru (S.S.K.); khanovam@gmail.com (M.Y.K.); sergtu@kemcardio.ru (T.Y.S.); matveeva_vg@mail.ru (V.G.M.); bio.tvg@mail.ru (T.V.G.); antonkutikhin@gmail.com (A.G.K.); rem57@rambler.ru (R.A.M.); deevanadusha69@yandex.ru (N.S.D.); t.akentyeva@mail.ru (T.N.A.); max-sinitsky@rambler.ru (M.Y.S.); velikanova_ea@mail.ru (E.A.V.); reception@kemcardio.ru (L.S.B.); 2 Budker Institute of Nuclear Physics of Siberian Branch Russian Academy of Sciences, 630090 Novosibirsk, Russia; vtkachen@mail.ru

**Keywords:** tissue-engineered vascular graft, preclinical development, ovine model, carotid artery, primary patency

## Abstract

Tissue-engineered vascular graft for the reconstruction of small arteries is still an unmet clinical need, despite the fact that a number of promising prototypes have entered preclinical development. Here we test Poly(3-hydroxybutyrate-co-3-hydroxyvalerate)Poly(ε-caprolactone) 4-mm-diameter vascular grafts equipped with vascular endothelial growth factor (VEGF), basic fibroblast growth factor (bFGF) and stromal cell-derived factor 1α (SDF-1α) and surface coated with heparin and iloprost (PHBV/PCL[VEGF-bFGF-SDF]^Hep/Ilo^, *n* = 8) in a sheep carotid artery interposition model, using biostable vascular prostheses of expanded poly(tetrafluoroethylene) (ePTFE, *n* = 5) as a control. Primary patency of PHBV/PCL[VEGF-bFGF-SDF]^Hep/Ilo^ grafts was 62.5% (5/8) at 24 h postimplantation and 50% (4/8) at 18 months postimplantation, while all (5/5) ePTFE conduits were occluded within the 24 h after the surgery. At 18 months postimplantation, PHBV/PCL[VEGF-bFGF-SDF]^Hep/Ilo^ grafts were completely resorbed and replaced by the vascular tissue. Regenerated arteries displayed a hierarchical three-layer structure similar to the native blood vessels, being fully endothelialised, highly vascularised and populated by vascular smooth muscle cells and macrophages. The most (4/5, 80%) of the regenerated arteries were free of calcifications but suffered from the aneurysmatic dilation. Therefore, biodegradable PHBV/PCL[VEGF-bFGF-SDF]^Hep/Ilo^ grafts showed better short- and long-term results than bio-stable ePTFE analogues, although these scaffolds must be reinforced for the efficient prevention of aneurysms.

## 1. Introduction

Despite immense efforts to reduce morbidity from atherosclerotic vascular disease [1] which have resulted in a steady decrease in the number of coronary artery bypass grafting procedures worldwide, this treatment modality remains common (82 procedures per 100,000 US adults annually) [2,3]. Vascular bypass implies the use of autologous blood vessel conduits (e.g., saphenous vein or internal mammary artery (IMA)) [4,5] while other types of arterial reconstruction involve biostable tubular scaffolds (e.g., Poly(ethylene terephthalate) (PET), expanded poly(tetrafluoroethylene) (ePTFE), or polyurethane prostheses) [6,7]. Yet, the limited availability of autografts (because of prior surgery, extensive atherosclerosis, or anatomical incompatibility) and high rate of thrombotic occlusion and neointimal hyperplasia in small diameter biostable prostheses limit their use in cardiovascular surgery [8,9]. Therefore, biodegradable, tissue-engineered vascular grafts (TEVGs) for the guided regeneration of vascular tissue have become a mainstream approach [8,9].

The fabrication of a commercially available off-the-shelf vascular graft requires excellent biocompatibility of its polymer composition for successful adhesion, migration, and proliferation of peripheral blood-derived cells, as well as to avert calcification [9,10,11]. Further, the appropriate prototype must possess a good haemocompatibility to prevent acute thrombosis and, ideally, should have a compliance profile similar with native coronary artery or IMA for precluding aneurysms and neointimal hyperplasia [9,10,11]. Current additive manufacturing technologies permit layer-by-layer incorporation of bioactive factors (e.g., growth factors or chemokines) to the tubular scaffold and their controlled release to guide endothelial/vascular smooth muscle specification and vascular tissue regeneration [11]. Rapid endothelialisation, propagation of contractile and synthetic mesenchymal cells (which can have smooth muscle or fibroblast identity), and production of the extracellular matrix (ECM) are key factors underlying high long-term primary patency of TEVGs [11,12,13].

Though natural polymers (e.g., collagen, elastin, or Poly(3-hydroxybutyrate-co-3-hydroxyvalerate, PHBV) generally have high biocompatibility, their durability is insufficient to resist the physiological blood pressure [11,12] that requires the addition of synthetic polymers (e.g., poly(ε-caprolactone), PCL) into the blend [14,15]. While endowing the scaffolds with superior mechanical properties, this manoeuvre may negatively affect cell differentiation. Together with turbulent flow conditions and low shear stress, increased rigidity of the grafts results in inadequate cyclic stretch and provokes a contractile-to-synthetic phenotype reprogramming of vascular smooth muscle cells, eventually leading to neointimal hyperplasia [16,17,18]. Moreover, synthetic vascular smooth muscle cells may further acquire osteochondrogenic specification that might lead to the development of calcifications which cause delamination of the extracellular matrix and are associated with the aneurysmatic dilation of the regenerated blood vessel [18,19]. Another shortcoming of adding synthetic polymers is an excessive infiltration of the graft by immune cells which thereby represent a majority of the cells within the regenerating blood vessel [20,21,22]. Further, the blending of natural and synthetic polymers does not improve haemocompatibility of the grafts, as rapid and complete endothelialisation of long vascular grafts, which is an important prerequisite to prevent thrombosis, is relatively rare [13,23,24]. To overcome this drawback, antiplatelet or anticoagulant drugs might be conjugated to a scaffold luminal surface [13].

Our group has previously designed an electrospun, small (4 mm) diameter PHBV/PCL vascular graft which employs the principle of guided vascular tissue regeneration through the release of a pro-angiogenic molecule vascular endothelial growth factor (VEGF) incorporated into the inner layer and a potent chemokine stromal cell-derived factor 1α (SDF-1α) incorporated into the outer layer along with another pro-angiogenic protein basic fibroblast growth factor (bFGF) [25,26,27]. To improve the hemocompatibility of this scaffold, here we attached an anticoagulant heparin (Hep) and a vasodilator/antiplatelet drug iloprost (Ilo) to its luminal surface (PHBV/PCL[VEGF-bFGF-SDF]^Hep/Ilo^). Biostable ePTFE vascular prostheses, which are frequently used in cardiovascular surgery, were selected as a control group [6,7]. For the proper assessment of the long-term primary patency and ultrastructural features, we used a sheep carotid artery interposition model since ovine arterial anatomy, haemodynamic conditions, and coagulation are similar to humans, while the long neck provides easy surgical access [28,29,30]. In addition, sheep exhibit accelerated rates of vascular calcification that allows evaluating the long-term prosthetic complications as soon as 1–2 years postimplantation [8].

In this study, we found that PHBV/PCL[VEGF-bFGF-SDF]^Hep/Ilo^ grafts demonstrated promising primary patency rate (62.5% and 50% at 24 h and 18 months postimplantation) in contrast to ePTFE conduits all of which were occluded within 24 h after the surgery. The regenerated arteries demonstrated a complete endothelialisation, high vascularisation and hierarchical multilayer structure reminiscent of the native blood vessels. Hence, PHBV/PCL[VEGF-bFGF-SDF]^Hep/Ilo^ grafts might be considered as a potential candidate for the further improvement and additional preclinical testing. However, PHBV/PCL[VEGF-bFGF-SDF]^Hep/Ilo^ grafts suffered from aneurysms, suggesting the need in their mechanical reinforcement.

## 2. Materials and Methods

### 2.1. Fabrication of TEVGs

Biodegradable TEVGs (4 mm diameter, ≈400 μm thickness and 4 cm length) were fabricated using emulsion electrospinning (Nanon-01A, MECC, Tokyo, Japan) from PHBV (403105, Sigma-Aldrich, Saint Louis, MO, USA): PCL (440744, Sigma-Aldrich, Saint Louis, MO, USA) (5:10%)/chloroform (366927, Sigma-Aldrich, Saint Louis, MO, USA) solution using the following parameters: 23 kV voltage, 0.5 mL/h feed rate, 2 mm rotating drum diameter, 22G needle, and 150 mm tip-to-collector distance. Abovementioned polymer ratio was determined in our previous studies [25,31,32]. In all these investigations, PHBV/PCL vascular grafts did not show any signs of dissolution as long as 1 year after implantation into rat abdominal aorta. Either VEGF (V7259, Sigma-Aldrich, St. Louis, MO, USA), bFGF (SRP4037, Sigma-Aldrich, St. Louis, MO, USA), or SDF-1α (SRP3276, Sigma-Aldrich, St. Louis, MO, USA) were dissolved in phosphate buffered saline (10010023, Thermo Fisher Scientific, Waltman, MA, USA) to 10 µg/mL concentration and then added to PHBV/PCL/chloroform solution (1:20), with the final concentration of 500 ng/mL. Grafts with the combination of VEGF, bFGF, and SDF-1α were two-layered, with the inner layer fabricated using 27G needle and containing VEGF (500 ng/mL) and the outer layer prepared utilizing 22G needle and containing bFGF and SDF-1α (500 ng/mL each).

### 2.2. Antithrombotic Modification of TEVG Luminal Surface

To increase the haemocompatibility of PHBV/PCL[VEGF-bFGF-SDF] vascular grafts, we first immobilised a PVP hydrogel at their luminal surface by incubating the prostheses in 5% alcoholic solution of PVP (K90, PanReac AppliChem, Darmstadt, Germany) for 30 min, drying at room temperature (20–24 °C) and sterile conditions for 24 h, and irradiating in the argon atmosphere by a linear particle accelerator (ILU-10, 50 kGy, electron energy 5 mEV, beam power 50 kW, Budker Institute of Nuclear Physics of Siberian Branch Russian Academy of Sciences, Novosibirsk, Russia). Grafts were then incubated in a 0.1 M glycine solution (pH = 2.6, 410225, Sigma-Aldrich, St. Louis, MO, USA) of heparin (125 IE/mL, Moscow Endocrine Plant, Moscow, Russia) and iloprost (0.2 μg/mL, Bayer, Barcelona-, Spain) for 30 min and dried at room temperature (20–24 °C) and sterile conditions for 24 h.

### 2.3. Evaluation of the Mechanical Properties

To evaluate the mechanical properties of TEVGs before and after Hep/Ilo immobilisation, uniaxial tension test was performed. Grafts were cut in the longitudinal axis using a custom-shaped knife in the Zwick/Roell cutting press (Zwick/Roell, Ulm, Germany). Segments of human internal mammary artery (length = 10 mm), excised during the coronary artery bypass grafting, were used for the control purposes. Tests were performed on the Z-series universal testing machine (Zwick/Roell, Ulm, Germany) with a nominal force of 50 N, limit of permissible error of ±1% and crosshead speed of 50 mm/min. We evaluated ultimate tensile strength, ultimate tensile force, elongation at break and elastic modulus; the latter was measured within the range of physiological pressure (80–120 mmHg). Prior to tensile testing, graft samples were not sterilised.

### 2.4. Haemocompatibility Testing

Assessment of haemolysis and platelet aggregation upon contact of the blood with a polymer surface was performed according to the ISO 10993.4 standard. For the haemolysis testing, fresh donor blood was collected into the improvacuter tubes (Guangzhou Improve Medical Instruments, Guangzhou, China) containing 3.8% sodium citrate. The citrate-to-blood ratio was 1:9. Polymer (PHBV/PCL[VEGF-bFGF-SDF], PHBV/PCL[VEGF-bFGF-SDF]^Hep/Ilo^, and ePTFE) samples (25 cm^2^, *n* = 5) were placed into a 10 mL physiological saline (0.9% NaCl solution, Binnofarm, Zelenograd, Russia) and incubated at 37 °C for 2 h to wet TEVGs for ensuring their proper interactions with red blood cells. Then, 200 mL citrated blood was added to each sample followed by repeated incubation at 37 °C for 1 h. Red blood cells were sedimented by centrifugation of the samples at 1446× *g* (2800 rpm, ELMI CM-6M, ELMI, Riga, Latvia) for 10 min. Optical density of the solution was measured using GENESYS 6 spectrophotometer (Thermo Scientific, Waltham, MA, USA) at 545 nm wavelength. Sample-induced haemolysis was reported as a percentage normalised to the positive control (double distilled water). Physiological saline was used as a negative control, as it is isotonic and therefore does not cause swelling or shrinking of red blood cells.

For the platelet aggregation analysis, we isolated platelet-rich plasma by the centrifugation of the blood at 184× *g* (1000 rpm, ELMI CM-6M, ELMI, Riga, Latvia) for 10 min. To measure platelet aggregation, we added 250 mL platelet-rich plasma to the polymer (PHBV/PCL[VEGF-bFGF-SDF], PHBV/PCL[VEGF-bFGF-SDF]^Hep/Ilo^, and ePTFE) samples (0.5 × 0.5 cm^2^) for 3 min and then added 25 μL 0.025 M CaCl_2_ (383147, Sigma-Aldrich, St. Louis, MO, USA) after removing the samples to activate platelet aggregation. Measurement was performed using APACT 4004 platelet aggregometer (LABiTec, Ahrensburg, Germany). Intact platelet-rich and platelet-poor plasma were used as a positive and negative control, respectively. Platelet-poor plasma was obtained by the centrifugation of platelet-rich plasma at 2260× *g* (3500 rpm, ELMI CM-6M, ELMI, Riga, Latvia) for 20 min.

### 2.5. Visualisation of TEVG Structure

An assessment of polymer (PHBV/PCL[VEGF-bFGF-SDF], PHBV/PCL[VEGF-bFGF-SDF]^Hep/Ilo^, and ePTFE) samples (0.5 × 0.5 cm^2^) was conducted following gold-palladium sputter coating (15 nm thickness, EM ACE200, Leica Microsystems, Wetzlar, Germany) by scanning electron microscopy (Hitachi S-3400N, Hitachi, Tokyo, Japan) in high vacuum mode at 10 kV voltage.

### 2.6. Animal Model

The study protocol was approved by the local ethical committee of the Research Institute for Complex Issues of Cardiovascular Diseases (protocol number 20180305, 28.04.2016, Kemerovo, Russia). Animal experiments were performed in accordance with the European Convention for the Protection of Vertebrate Animals (Strasbourg, 1986). For the in vivo experiments, we used female Edilbay sheep of 42–45 kg body weight which were received from the Animal Core Facility of the Research Institute for Complex Issues of Cardiovascular Diseases (Kemerovo, Russia). Sheep were selected for the surgery by Doppler ultrasonography to identify those having carotid artery diameter of 3.8–4.2 mm. Among the tested samples were biodegradable PHBV/PCL[VEGF-bFGF-SDF]^Hep/Ilo^ TEVGs (*n* = 8, 7 grafts implanted for 18 months and 1 graft implanted for 6 months to evaluate a biodegradation rate) and biostable ePTFE vascular prostheses (*n* = 5, all implanted for 6 months, GORE-TEX, ST04010A, W. L. Gore and Associates, Newark, DE, USA).

Anaesthesia was induced with zoletil (Virbac, Carros, France) and maintained by artificial ventilation with sevoflurane (Baxter International, Deerfield, IL, USA) with constant control of heart rate, respiratory rate, and oxygen saturation. Upon achieving access to the carotid artery and intravenous infusion of heparin (5000 IU), we clamped the artery, excised a 4 cm segment, performed end-to-end implantation of a vascular prosthesis using the twisted seam (Prolene 6-0, Ethicon, Somerville, NJ, USA), and conducted the wound closure (Vicryl 2-0, Ethicon, Somerville, NJ, USA). Postoperative care included daily infusions of cefuroxime (Medochemie, Limassol, Cyprus). Graft patency was assessed by Doppler ultrasonography immediately after the surgery and then at the following time points: 24 h, 3 months, 6 months, 12 months, and 18 months postoperation.

### 2.7. Histological, Immunofluorescent, and Ultrastructural Examination

At 6 or 18 months postoperation, sheep were sacrificed. At the site of the implantation, the graft (or the regenerated artery with the scaffold remnants) was excised and cut into four segments of the equal length. 

The first segment was fixed in two changes of 10% neutral phosphate buffered formalin (B06-003, BioVitrum, St. Petersburg, Russia) for 24 h at 4 °C, dehydrated in ascending ethanol series (70, 80, and 95%, 1 h per each) and isopropanol (1 h, 06-002, BioVitrum, St. Petersburg, Russia), impregnated and embedded into paraffin (56 °C, 3 changes, 1 h per each, Paraplast REGULAR, 39601006, Leica), cooled at 4 °C overnight and cut (5 µm sections) on a microtome (Microm HM 325, Thermo Scientific, Waltham, MA, USA). To ensure the proper histological examination, we prepared 12 sections, evenly distributed across the entire excised segment, per slide. Upon the deparaffinisation in three changes of xylene (23400, Electron Microscopy Sciences, Hatfield, PA, USA) and three changes of 95% ethanol, sections were stained with: (1) haematoxylin and eosin (ab245880, Abcam, Cambridge, UK) according to the manufacturer’s protocol for the general examination; (2) van Gieson stain (21-020, BioVitrum, St. Petersburg, Russia) as in [31] to distinguish connective and smooth muscle tissue; (3) 2% aqueous alizarin red S (ab146374, Abcam, Cambridge, UK) and DAPI (10 μg/mL, D9542, Sigma-Aldrich, St. Louis, MO, USA) as in [31] for the detection of calcium deposits within the grafts. Visualisation was performed by light or fluorescent microscopy (AxioImager.A1, Carl Zeiss, Oberkochen, Germany).

The second segment was snap-frozen in the optimal cutting temperature medium (Tissue-Tek, 4583, Sakura Finetek, Tokyo, Japan) and cut on a cryostat (Microm HM 525, Thermo Scientific, Waltham, MA, USA) as described above. Sections (5 µm thickness) were then stained with rabbit anti-CD31 (ab28364, Abcam, Cambridge, UK) and mouse anti-α-SMA (ab7817, Abcam, Cambridge, UK); or rabbit anti-vWF (ab6994, Abcam, Cambridge, UK); or rabbit anti-Collagen IV (ab6586, Abcam, Cambridge, UK) and mouse anti-Collagen I (ab23446, Abcam, Cambridge, UK); or rabbit anti-collagen III (NB600-594, Novus Biologicals, Centennial, CO, USA) primary antibodies. Samples were further treated with goat antirabbit highly cross-adsorbed Alexa Fluor 488-conjugated (A11034, Thermo Fisher Scientific, Waltham, MA, USA) and donkey antimouse highly cross-adsorbed Alexa Fluor 555-conjugated (A31570, Thermo Fisher Scientific, Waltham, MA, USA) secondary antibodies. Counterstaining was performed with DAPI (10 μg/mL, 30 min, D9542, Sigma-Aldrich, St. Louis, MO, USA). Visualisation was performed by a confocal microscopy (LSM700, Carl Zeiss, Oberkochen, Germany).

The third segment was fixed in two changes of 10% neutral phosphate buffered formalin for 24 h at 4 °C, postfixed in 1% phosphate buffered osmium tetroxide (OsO4, 19110, Electron Microscopy Sciences, Hatfield, PA, USA) for 24 h, stained in 2% aqueous osmium tetroxide for 48 h, dehydrated in ascending ethanol series (50, 60, 70, 80 and 95%, 15 min per each), stained in 2% alcoholic uranyl acetate (22400-2, Electron Microscopy Sciences, Hatfield, PA, USA) for 5 h, dehydrated in isopropanol for 5 h and acetone (1 h), impregnated with acetone: epoxy resin (Epon, 14120, Electron Microscopy Sciences, Hatfield, PA, USA) mixture (1:1) for 6 h and with epoxy resin for 24 h, and finally embedded into the fresh epoxy resin at 60 °C. Samples were then ground, polished (TegraPol-11, Struers, Copenhagen, Denmark), and counterstained with Reynolds’s lead citrate (17810, Electron Microscopy Sciences, Hatfield, PA, USA) for 15 min. After a brief washing in double distilled water, samples were sputter coated (10 nm thickness) with carbon (EM ACE200, Leica, Wetzlar, Germany) and visualised by means of backscattered scanning electron microscopy at 10 or 15 kV voltage (S-3400N, Hitachi, Tokyo, Japan). Elemental analysis was carried out by energy-dispersive X-ray spectroscopy (XFlash 4010, Bruker, Billerica, MA, USA) in a low vacuum mode at 15 kV voltage.

### 2.8. Transcriptional Profiling

At 18 months postimplantation, the fourth segment of the regenerated carotid arteries replacing the TEVG, and intact contralateral carotid arteries was flushed with TRIzol Reagent (15596018, Thermo Fisher Scientific, Waltham, MA, USA) to collect endothelial RNA. Then, de-endothelialised blood vessels were homogenised in TRIzol Reagent (FastPrep-24 Instrument and Lysing Matrix S, 116925050-CF, MP Biomedicals, Irvine, CA, USA) to extract the remaining RNA of other vascular cell populations. Upon the RNA isolation according to the manufacturer’s protocol, reverse transcription (RT) was carried out utilising High-Capacity cDNA Reverse Transcription Kit (4368814, Thermo Fisher Scientific, Waltham, MA, USA). Gene expression was measured by a quantitative polymerase chain reaction (RT-qPCR) using the customised primers (Table 1). Primers were produced using ABI 3900 high-throughput DNA synthesiser (Thermo Fisher Scientific, Waltham, MA, USA) at Evrogen (Moscow, Russia). To perform RT-qPCR, primers (500 nmol/L each), cDNA (20 ng) and PowerUp SYBR Green Master Mix (A25778, Thermo Fisher Scientific, Waltham, MA, USA) were mixed and incubated according to the manufacturer’s protocol for Tm ≥ 60 °C (fast cycling mode). Technical replicates (*n* = 3 per each sample of the endothelial lysate or homogenate of the de-endothelialised vascular tissue) were performed in all RT-qPCR experiments. The reaction was considered successful if its efficiency was 90–105% and R2 was ≥0.98. Quantification of the mRNA levels (IL1B, IL6, CXCL8, ICAM1, MMP2, KDR, NR2F2, and SNAI2 genes) in the indicated samples was performed by using the 2^−ΔΔCt^ method. Relative transcript levels in the endothelial lysate or de-endothelialised vascular tissue within the regenerated artery replacing the TEVG were expressed as a value relative to the average of two housekeeping genes (GAPDH and B2M) and to the respective RNA fractions of the intact contralateral carotid artery (2^−ΔΔCt^). Alternatively, relative transcript levels in the endothelial lysate of the regenerated artery were expressed as a value relative to the same housekeeping genes and to the corresponding de-endothelialised vascular tissue. The adjusted values were finally represented as a heat map (green, grey and red colours reflected fold change ≤0.50, 0.51–1.99, and ≥2.00, respectively).

### 2.9. Statistical Analysis

Statistical analysis was performed using GraphPad Prism 8 (GraphPad Software, San Diego, CA, USA). Data were presented as the median and interquartile range. Independent groups were compared using Kruskal-Wallis test with Dunn’s multiple comparisons test. *p* values of ≤0.05 were regarded as statistically significant.

## 3. Results

### 3.1. Hep/Ilo-Treated TEVGs Are Mechanically Competent

To demonstrate an adequate performance within the living organism, TEVGs are required to withstand an initial hydrodynamic pressure burst (i.e., to show a sufficient durability) and to properly adapt to the repeated changes of the blood pressure occurring during the cardiac cycle (i.e., to exhibit a considerable elasticity). We first conducted a tensile testing of PHBV/PCL[VEGF-bFGF-SDF]^Hep/Ilo^ grafts to reveal whether the Hep/Ilo attachment affects their mechanical properties. As a kind of control, we used human internal mammary artery which is often applied for the arterial replacement [5]. Stiffness (reflected by the elastic modulus) of PHBV/PCL[VEGF-bFGF-SDF]^Hep/Ilo^ grafts were 6- and 21-fold higher than in unmodified PHBV/PCL[VEGF-bFGF-SDF] prostheses and IMA, respectively (Table 2). Likewise, chemical immobilisation of heparin and iloprost increased durability but slightly reduced elasticity of TEVGs which still exceeded those of IMA (Table 2). Expectedly, the durability and elasticity of the biostable ePTFE prostheses was significantly higher than in TEVGs and IMA, whereas their stiffness was close to that of the latter conduit (Table 2).

### 3.2. Conjugation of Hep/Ilo with the Luminal Surface of TEVGs Improves Their Haemocompatibility

Haemocompatibility is a key factor in providing short-term patency of the graft [24,33]. Thrombotic occlusion is primarily caused by a platelet aggregation [24,33] which has been measured upon the contact of platelet-rich plasma with unmodified PHBV/PCL[VEGF-bFGF-SDF] grafts or PHBV/PCL[VEGF-bFGF-SDF]^Hep/Ilo^ grafts having an antithrombotic coating. Control PHBV/PCL[VEGF-bFGF-SDF] grafts induced a considerable aggregation of platelets similar to the intact platelet-rich plasma upon the calcium chloride activation (Table 2). Immobilisation of heparin and iloprost at the luminal surface resulted in a 2–3 fold decrease in platelet aggregation (Table 3). Haemolysis did not exceed 0.4% in all experimental groups testifying to the high haemocompatibility of PHBV/PCL[VEGF-bFGF-SDF]^Hep/Ilo^ grafts (Table 3).

### 3.3. Immobilisation of Hep/Ilo at the Luminal Surface of TEVGs by Poly(N-vinylpyrrolidone) Preserves Their Structure

**Figure 1:**
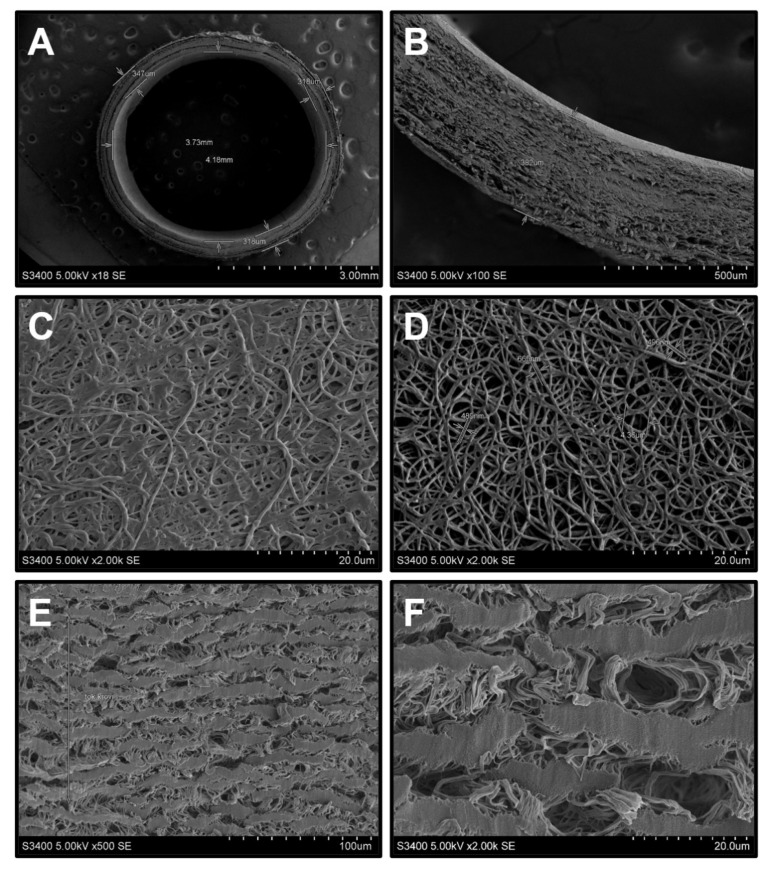
Scanning electron microscopy of biodegradable and synthetic vascular grafts: (**A**) Layer-by-layer hierarchical structure of the PHBV/PCL[VEGF-bFGF-SDF]^Hep/Ilo^ grafts, view from above, ×18 magnification; (**B**) Layer-by-layer hierarchical structure of the PHBV/PCL[VEGF-bFGF-SDF]^Hep/Ilo^ grafts, side view, ×100 magnification; (**C**) Luminal surface of the PHBV/PCL[VEGF-bFGF-SDF]^Hep/Ilo^ grafts before the washing from PVP, ×2000 magnification; (**D**) Luminal surface of the PHBV/PCL[VEGF-bFGF-SDF]^Hep/Ilo^ grafts after the washing from PVP, ×2000 magnification; (**E**) Luminal surface of ePTFE tubular scaffold, ×500 magnification; (**F**) Luminal surface of ePTFE tubular scaffold, ×2000 magnification. Representative images.

Structurally, electrospun PHBV/PCL[VEGF-bFGF-SDF]^Hep/Ilo^ scaffolds represented a layered microfiber network (Figure 1A,B) which consisted of multiple interconnected pores (Figure 1C,D). Binding of Hep/Ilo to the luminal surface of the PHBV/PCL[VEGF-bFGF-SDF] grafts is achieved via its hydrophilic coating with poly(N-vinylpyrrolidone) (PVP) hydrogel which also occupies the pores (Figure 1C) and prevents protein adsorption [34], potentially being one of the factors behind the reduced induction of platelet aggregation by PHBV/PCL[VEGF-bFGF-SDF]^Hep/Ilo^ grafts. Experimental washing from PVP remained the pores intact (Figure 1D), confirming retained integrity of the prostheses upon the chemical immobilisation of heparin and iloprost. In contrast to TEVGs, biostable prostheses were composed of wide and thick ePTFE fragments and large pores (Figure 1E,F).

### 3.4. Hep/Ilo Coating Endows TEVGs with a Considerable Long-Term Primary Patency

**Figure 2:**
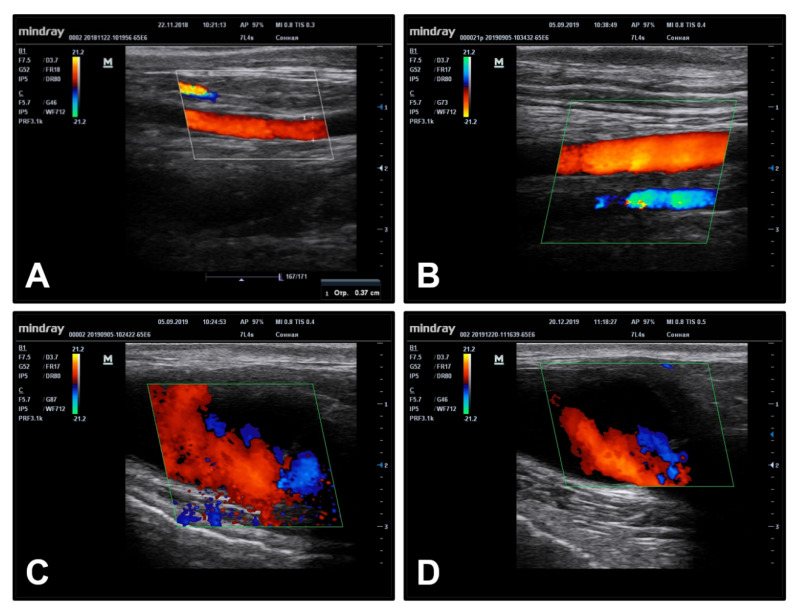
Time-resolved Doppler ultrasound images of the PHBV/PCL[VEGF-bFGF-SDF]^Hep/Ilo^ grafts implanted into the sheep carotid artery. (**A**) 24 h postimplantation; (**B**) 3 months postimplantation; (**C**) 6 months postimplantation; (**D**) 18 months postimplantation. Representative images.

We next implanted PHBV/PCL[VEGF-bFGF-SDF]^Hep/Ilo^ grafts (*n* = 8) into the sheep carotid artery to test their haemo- and bio- compatibility in terms of primary patency which was assessed at 24 h, 3, 6, 12, and 18 months postimplantation by Doppler ultrasonography. Short-term (24 h postimplantation, Figure 2A) and mid-term (3 and 6 months postimplantation, Figure 2B,C) patency of PHBV/PCL[VEGF-bFGF-SDF]^Hep/Ilo^ grafts was 65% (5 out of 8). At 12 months postimplantation, one of the scaffolds became occluded. Hence, long-term (18 months postimplantation) primary patency of PHBV/PCL[VEGF-bFGF-SDF]^Hep/Ilo^ grafts was 50% (4/8) (Figure 2D).

**Figure 3:**
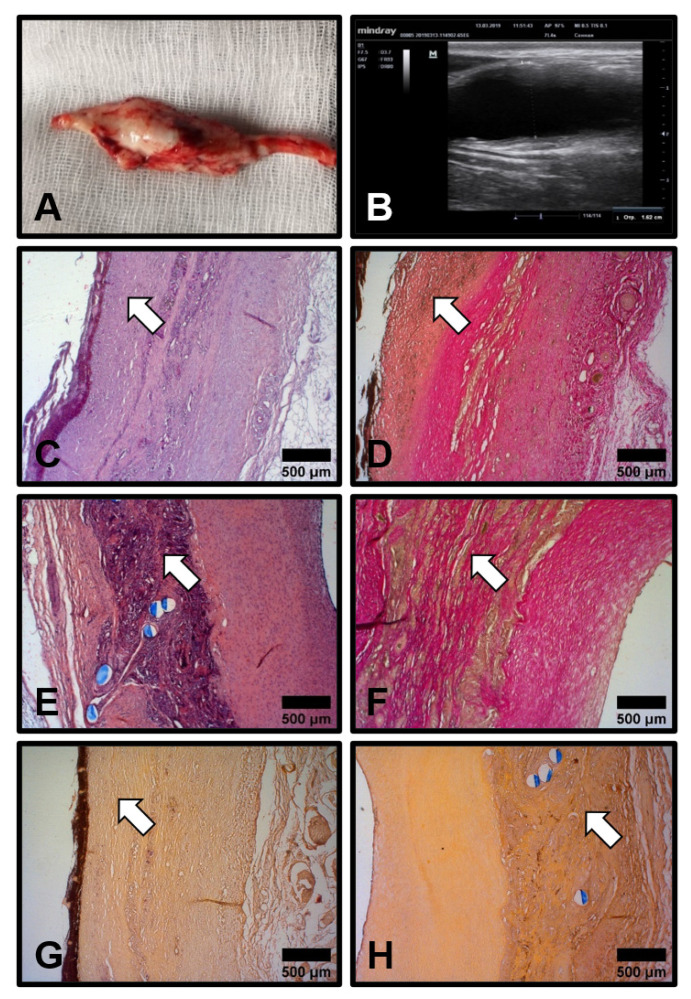
Development of the aneurysms in the patent PHBV/PCL[VEGF-bFGF-SDF]^Hep/Ilo^ grafts 6 months postimplantation. (**A**) Gross examination of the aneurysm which developed through the whole length of the graft; (**B**) Ultrasound examination of the same aneurysm; (**C**,**D**) Neointima (indicated by white arrows) demarcated from the degrading polymer scaffold by an organised layer of collagen fibres. Haematoxylin and eosin staining (**C**) and van Gieson staining (**D**), ×50 magnification; (**E**,**F**) Degrading polymer scaffold is partially substituted by collagen bundles (white arrows) formed de novo. Haematoxylin and eosin staining (**E**) and van Gieson staining (**F**), ×50 magnification; (**G**,**H**) Absence of calcium deposits both within the neointima (**G**, white arrow) and polymer scaffold (**H**, white arrow). Alizarin red S staining, ×50 magnification. Representative images.

At 6 months postimplantation, the diameter of 80% (4/5) of the patent PHBV/PCL[VEGF-bFGF-SDF]^Hep/Ilo^ grafts increased from 4 to 22 mm, which was indicative of aneurysm formation (Figure 3A,B). The remaining graft without an aneurysm was stenosed and characterised by a low blood flow. A histological examination of the aneurysmatic grafts revealed a neointima (Figure 3C,D) and large amounts of regenerated vascular tissue which was particularly prominent in the medial layer (Figure 3E,F), suggesting an ongoing replacement of the PHBV/PCL scaffold. Resorption of the prosthetic tissue was confirmed by the abundant connective tissue around the polymer fibres (Figure 3D,F). No signs of calcium deposition have been revealed both in the neointima and within the degrading polymer scaffold (Figure 3G,H). The rapid resorption of the scaffold contrasted to both data described in the literature (2–3 years for PCL) [35,36] and our previous results from a rat aortic interposition model [25,26,27]. The development of the aneurysms indicated the immaturity of the regenerated artery.

### 3.5. Cellular and Molecular Composition of PHBV/PCL[VEGF-bFGF-SDF]^Hep/Ilo^ Grafts Is Reminiscent of Native Arteries

**Figure 4:**
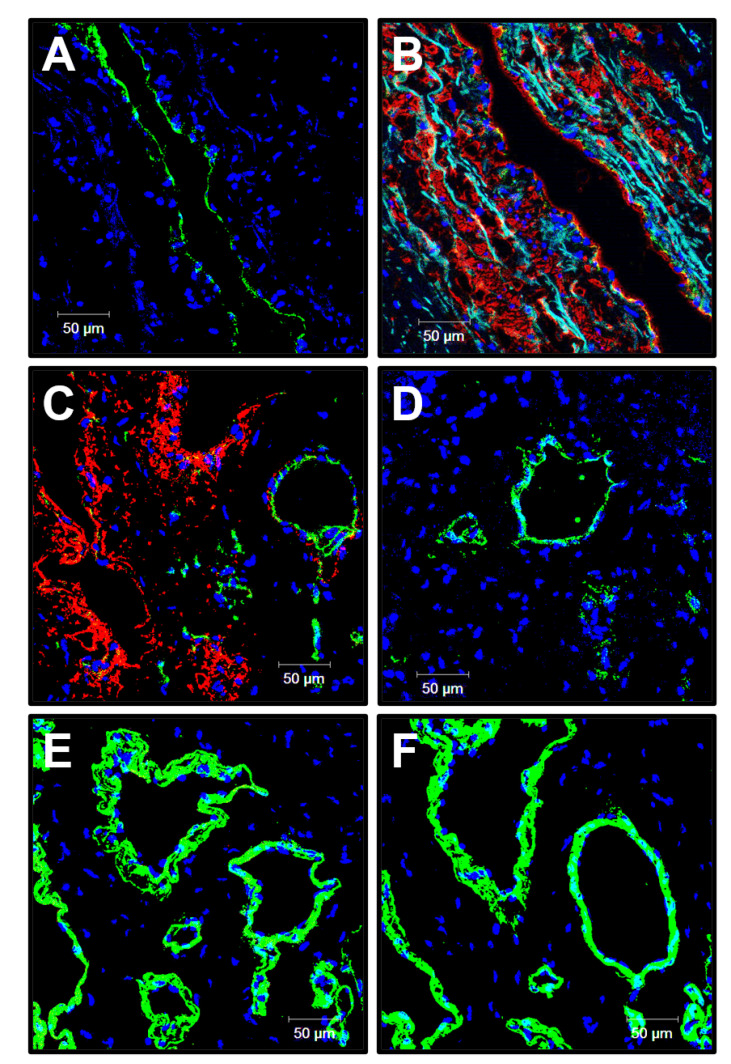
Confocal microscopy examination of the PHBV/PCL[VEGF-bFGF-SDF]^Hep/Ilo^ graft 6 months postimplantation. (**A**) CD31 (a marker of endothelial cells, green colour) staining; (**B**) α-SMA (a marker of VSMCs, red colour) staining; (**C**) Combined CD31 (green colour) and-SMA (red colour) staining of the vasa vasorum within the graft; (**D**) vWF (a marker of endothelial cells and a component of the subendothelial ECM, green colour) staining of the vasa vasorum within the graft; (**E**) Type IV collagen (an ECM protein constituting the vascular basement membrane, green colour) staining; (**F**) Type III collagen (an ECM protein constituting the vascular basement membrane, green colour) staining. 4′,6-diamidino-2-phenylindole (DAPI) counterstaining (nuclei, blue colour). Representative images, ×200 magnification.

To better examine the cell populations within the patent PHBV/PCL[VEGF-bFGF-SDF]^Hep/Ilo^ grafts and structure of the aneurysmatic ECM 6 months postimplantation, we stained excised vascular prostheses for endothelial markers CD31 and von Willebrand factor (vWF), vascular smooth muscle cell (VSMC) marker α-smooth muscle actin (α-SMA), and different types of arterial collagen. The examined grafts were well endothelialised (CD31+ cells at the luminal surface, Figure 4A), contained multiple layers of VSMCs in the medial layer (Figure 4B), and were highly vascularised (multiple vasa vasorum, Figure 4C–F). Similar to the large arteries [37], the microvessels within the graft were positively stained for CD31 and α-SMA (Figure 4C), vWF (Figure 4D) as well as type IV (Figure 4E) and type III collagen (Figure 4F) composing the vascular basement membrane.

**Figure 5:**
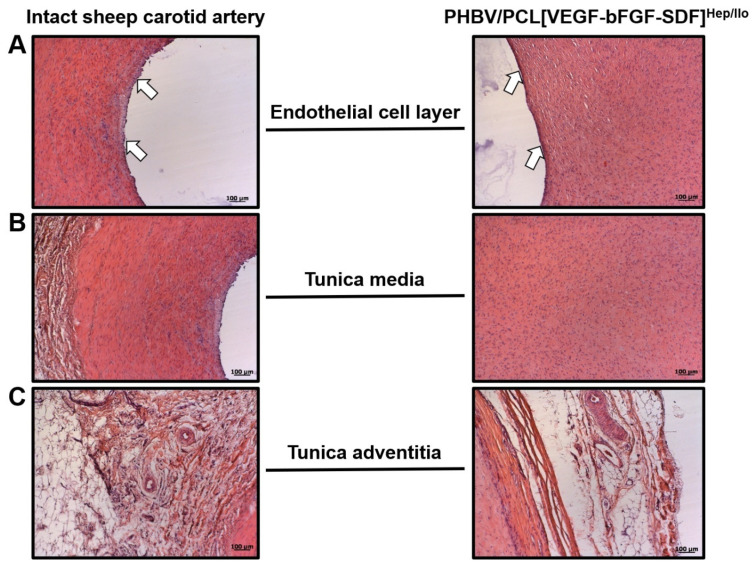
Histological comparison of the intact ovine carotid artery and regenerated artery which replaced PHBV/PCL[VEGF-bFGF-SDF]^Hep/Ilo^ graft 18 months postimplantation. (**A**) Endothelial cell layer (indicated by white arrows); (**B**) Tunica media; (**C**) Tunica adventitia and perivascular adipose tissue. Haematoxylin and eosin staining, representative images, ×100 magnification.

At 18 months postimplantation, the PHBV/PCL scaffold was almost resorbed and replaced by a blood vessel which consisted of three layers similar to a contralateral carotid artery. Typically, regenerated arteries were endothelialised (Figure 5A), retained their integrity (Figure 5B), and had ample connective tissue and numerous vasa vasorum in the outer layer resembling the tunica adventitia (Figure 5C). Perivascular adipose tissue was also observed (Figure 5C). However, regenerated arteries were notable for the absence of both elastic fibres and hierarchical orientation of the VSMCs (Figure 5B) that was the most probable cause of the aneurysms.

**Figure 6:**
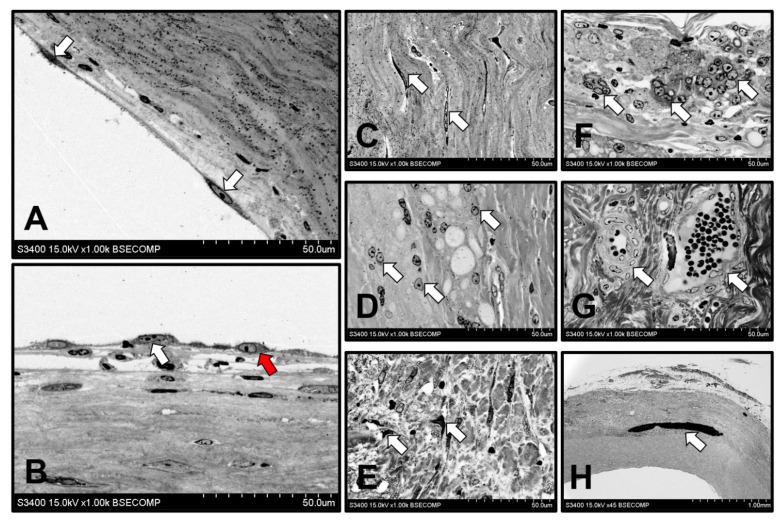
Backscattered scanning electron microscopy examination of the regenerated artery which replaced PHBV/PCL[VEGF-bFGF-SDF]^Hep/Ilo^ graft 18 months postimplantation. (**A**) Monolayer of elongated endothelial cells (indicated by white arrows), ×1000 magnification; (**B**) Combination of elongated endothelial cells (white arrow) and polymorphic endothelial cells (red arrow) suggestive of a transitional phenotype, ×1000 magnification; (**C**) VSMCs in the neointima (white arrows), ×1000 magnification; (**D**) Macrophages in the medial layer (white arrows) which substituted a polymer scaffold, ×1000 magnification; (**E**) Fibroblast-like cells in the medial layer (white arrows), ×1000 magnification; (**F**) Multinucleated giant cells in the tunica adventitia (white arrows), ×1000 magnification; (**G**) Adventitial vasa vasorum (white arrows), ×1000 magnification; (**H**) Calcification on the border of neointimal and medial layers (white arrow), ×45 magnification. Representative images.

We then performed an ultrastructural investigation of the regenerated arteries by means of backscattered scanning electron microscopy. Neointima, media, and adventitia were clearly identified. Endothelial cells were either elongated in the direction of flow (Figure 6A) or acquired a polymorphic shape (Figure 6B), possibly owing to the endothelial-to-mesenchymal transition. Neointima consisted of VSMCs confined in the dense ECM (Figure 6C), while the loose medial layer which replaced a resorbable scaffold was populated by macrophages (Figure 6D) and fibroblast-like cells (Figure 6E). Tunica adventitia contained the loose ECM, numerous multinucleated giant cells (Figure 6F), and vasa vasorum (Figure 6G) formed during the polymer resorption.

**Figure A1:**
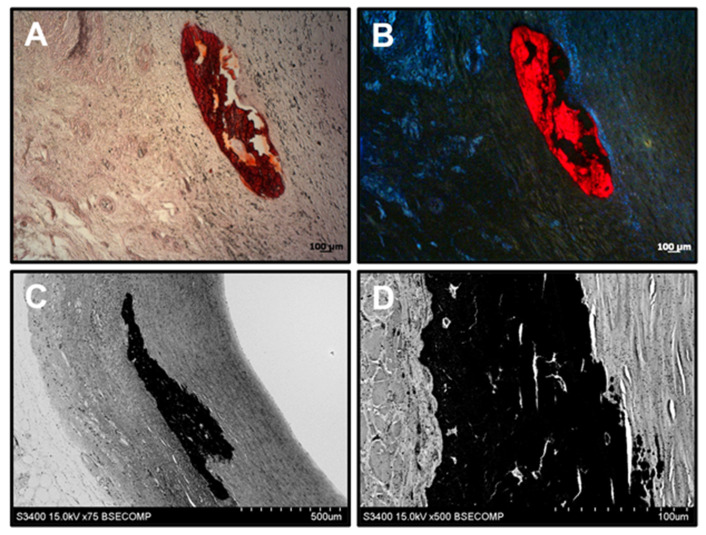
Calcification of a regenerated artery on the border of neointimal and medial layers 18 months postimplantation. (**A**) Alizarin red S staining, light microscopy, ×50 magnification; (**B**) Alizarin red S staining with DAPI counterstaining, ×50 magnification; (**C**) Backscattered scanning electron microscopy, ×75 magnification; (**D**) Backscattered scanning electron microscopy, ×500 magnification.

One of the most hazardous long-term complications of TEVG implantation is calcification, which frequently leads to a delamination of the regenerated vessel and pseudoaneurysm formation [38,39]. Despite the fact that the ovine model is considered to be the “worst case scenario”, as sheep are prone to the mineralisation of cardiovascular implants [8], we did not observe calcification loci in PHBV/PCL[VEGF-bFGF-SDF]^Hep/Ilo^ grafts 6 months postimplantation. At the 18-month time point, one of the regenerated arteries (1/5, 20%) contained a single mineral deposit which had structural and chemical homogeneity (Figure 6H and Figure A1). The calcium to phosphorus ratio of 2.08 was indicative of carbonate-hydroxyapatite (bioapatite) [40,41].

**Figure 7:**
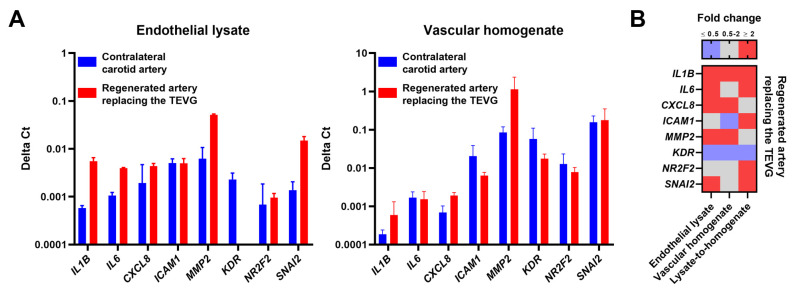
Transcriptional profiling of the regenerated and intact contralateral carotid arteries 18 months postimplantation. (**A**) Delta Ct values for the measured transcripts (IL1B, IL6, CXCL8, ICAM1, MMP2, KDR, NR2F2 and SNAI2) in the endothelial lysate (left) and homogenate of the de-endothelialised blood vessel (right); (**B**) Heat map indicating the differences between the endothelial lysate and vascular homogenate of the regenerated artery in relation to the respective RNA fractions in the contralateral intact artery and between endothelial lysate and homogenate of the de-endothelialised regenerated artery normalised to the mRNA expression in these compartments in the contralateral intact artery. Blue, grey and red colours mean fold change ≤0.50, 0.51–1.99, and ≥2.00, respectively, compared to the contralateral intact artery or homogenate of the de-endothelialised regenerated artery.

Next, we compared the transcriptional profiles of the endothelium and the wall of the regenerated artery in relation to those in contralateral carotid arteries. The excised vessels were flushed with TRIzol to collect the endothelial lysate and further homogenised to extract the RNA from the vascular wall. Reverse transcription-quantitative polymerase chain reaction revealed the abundance of the transcripts associated with inflammation (IL1B, IL6, and CXCL8), ECM remodelling (MMP2) and endothelial-to-mesenchymal transition (SNAI2) in both RNA fractions obtained from the regenerated artery (Figure 7A,B). Endothelial lysate was enriched with the inflammatory transcripts (IL1B, IL6, and ICAM1) and signatures of endothelial reprogramming (a venous transcript NR2F2 and an endothelial-to-mesenchymal transition marker SNAI2) (Figure 7B). These observations suggest that the molecular landscape of TEVGs can differ from the corresponding blood vessels, even with long-term arterial regeneration (18 months postimplantation).

### 3.6. Biostable ePTFE Vascular Prostheses Undergo Thrombotic Occlusion and Calcification

**Figure 8:**
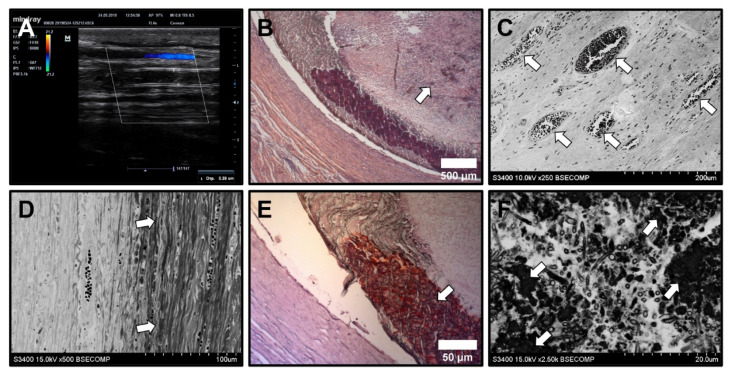
Histological and ultrastructural examination of ePTFE vascular prostheses. (**A**) Detection of thrombosis by Doppler ultrasound examination; (**B**) Thrombotic occlusion (white arrow), haematoxylin and eosin staining, ×50 magnification; (**C**) Recanalised thrombus (recanalising microvessels are indicated by white arrows), backscattered scanning electron microscopy, ×250 magnification; (**D**) Connective tissue capsule (white arrows), backscattered scanning electron microscopy, ×500 magnification. (**E**) Calcification (white arrow) within the connective tissue capsule, alizarin red S staining, ×100 magnification; (**F**) Microcalcifications within the thrombus (white arrows), backscattered scanning electron microscopy, ×250 magnification. Representative images.

In contrast to PHBV/PCL[VEGF-bFGF-SDF]^Hep/Ilo^ grafts, ePTFE vascular prostheses were occluded as early as 24 h postimplantation (Figure 8A,B). At 6 months postimplantation, thrombosis became recanalised (Figure 8C) and biostable grafts were surrounded by a connective tissue capsule (Figure 8D) which was calcified (Figure 8E), suggesting a foreign body reaction. Multiple microcalcifications were also detected within the thrombus (Figure 8F). This histopathological pattern significantly differed from that observed in TEVGs.

**Figure 9:**
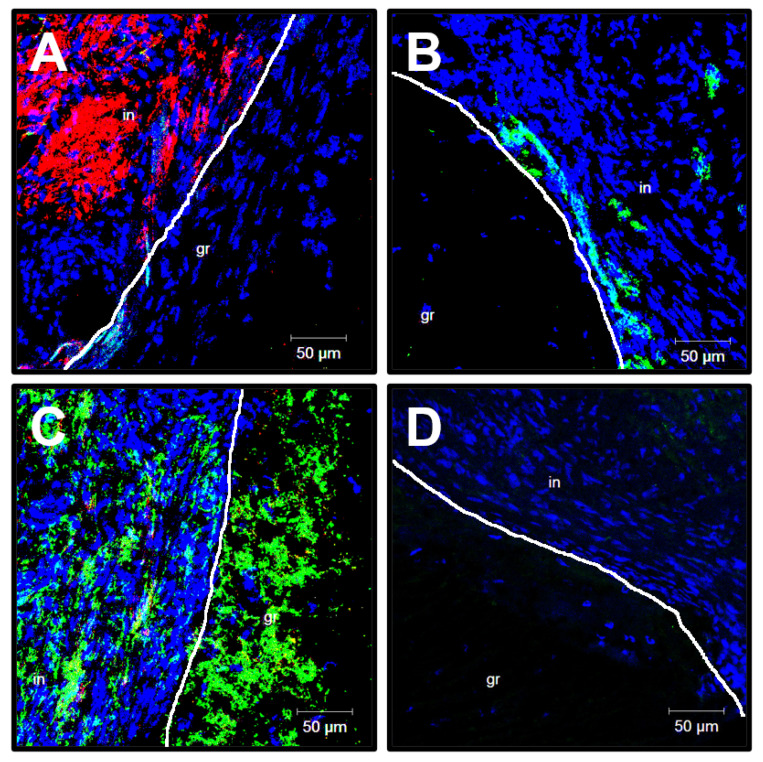
Confocal microscopy examination of ePTFE vascular prostheses 6 months postimplantation. (**A**) CD31 (a marker of endothelial cells, green colour) and α-SMA (a marker of VSMCs, red colour) staining; (**B**) von Willebrand staining (a marker of endothelial cells and a component of the subendothelial ECM, green colour); (**C**) Type IV collagen (an ECM protein constituting the basement membrane, green colour) and type I collagen (an ECM protein characteristic of the adventitia, red colour) staining; (**D**) Type III collagen staining (an ECM protein constituting the basement membrane and tunica media, green colour). DAPI counterstaining (nuclei, blue colour). White line demarcates the graft (gr) from thrombotic masses (in). Representative images, ×200 magnification.

Immunofluorescent examination of ePTFE grafts at 6 months postimplantation revealed the absence of the endothelium suggestive of poor biocompatibility (CD31- and largely vWF-negative cells at the luminal surface, Figure 9A,B). VSMCs were identified within the thrombus but not in the medial layer (Figure 9A). Disorganised bundles of type IV collagen were located beneath the luminal surface (Figure 9C) whereas type III and type I collagen were not found (Figure 9C,D).

## 4. Discussion

Despite the fact that numerous rigorous studies and preclinical trials have been performed over the past decade, a commercially available, off-the-shelf TEVG has not been developed to date. The reasons for this include insufficient durability of the prototypes fabricated from biocompatible natural polymers, poor biocompatibility of synthetic polymers, and uncontrollable biodegradation of polymer blends [9,10,11,12,13,42]. Further, small animal models are inappropriate for TEVG testing, as the haemodynamic conditions in the blood vessels and parameters of haemostasis in mice and rats are vastly different from those in humans [28,29,30]. Yet, the use of large animals such as dogs, swine, or sheep is complicated because of their high cost and difficult handling [28,29,30]. Among the available animal models, haemostasis and inflammatory response in sheep show the highest similarity to humans, while their carotid artery is sufficiently long for proper mechanical testing and is easily accessible for the surgical intervention and postimplantation visualisation [28,29,30]. Sheep are also characterised by an accelerated calcification of cardiovascular implants mirroring the clinical scenario in working-age patients [8]. Hence, we chose an ovine carotid artery interposition model for the animal testing of our TEVGs which were previously investigated in rats to verify the positive effects of incorporated growth factors on vascular tissue regeneration [25,26,27].

Implantation of a TEVG into the circulatory system is often accompanied by a number of significant complications. The first is thrombosis, which occurs within the first 24 h postimplantation and often during surgery in cases of contact activation induced by a negatively charged polymer surface [9,12,33]. Long-term pathological consequences include: (1) neointimal hyperplasia which leads to the restenosis and develops due to the proliferation of mesenchymal cells (i.e., VSMCs and fibroblasts), active synthesis of the ECM, and concurrent vascularisation-driven inflammation; (2) calcification, which provokes delamination and causes mechanical incompetence of the graft, emerging as a result of an uncontrolled degradation of the polymer fibres, incomplete or improper assemble of the ECM and osteogenic differentiation of mesenchymal cells; (3) aneurysms, which may induce graft rupture and occur because of dyscoordinated polymer replacement with the regenerating ECM and uneven distribution of the mechanical load across the graft [12,33].

We previously demonstrated the high primary patency rate of an electrospun scaffold prepared from PHBV/PCL blend enriched with VEGF, bFGF and SDF-1α by using a rat abdominal aorta replacement model [26,27]. The applied electrospinning technique generated nanoscale polymer fibres which formed interconnected microsized pores, thereby increasing the area for cell-ECM interactions [11,43]. Combined with bioactive factors, such construction of the conduit potentiated migration, attachment, and guided differentiation of blood-derived cells, consequently improving the integration of the implant with the host tissues [32]. However, preliminary studies found unacceptably high frequency of thrombosis when this prototype was applied to replace ovine carotid artery. To solve this issue, we immobilised Hep/Ilo on a luminal surface through the PVP linker to endow the graft with antithrombotic properties before the endothelialisation. In this study, we tested the aforementioned TEVG (PHBV/PCL[VEGF-bFGF-SDF]^Hep/Ilo^) against the biostable ePTFE vascular prosthesis, which is not replaced by de novo ECM and has superior mechanical properties but exhibits poor endothelialisation.

The majority (62.5%) of the PHBV/PCL[VEGF-bFGF-SDF]^Hep/Ilo^ grafts, but none of the ePTFE prostheses, showed absence of thrombosis, and 50% of TEVGs were patent 18 months postimplantation, providing data of future improvements of this prototype. However, in contrast to ePTFE grafts, almost all regenerated arteries (i.e., vascular tissue which replaced the grafts) suffered from aneurysms. The reason behind this was the unexpectedly [35,36] rapid resorption of the PHBV/PCL scaffold, which lost its integrity 6 months postimplantation and was almost completely resorbed 18 months postimplantation. In rats, all grafts were intact 12 months postimplantation and did not display any signs of aneurysm formation [27,32]. Intriguingly, these results fully correspond to a recent paper which also reported a striking difference in the degradation rates of TEVGs between small and large animal models [44]. Similar to our previous and present research, this study employed rat abdominal aorta and sheep carotid artery interposition models for the implantation of PCL/chitosan vascular graft [44]. In keeping with the abovementioned results, ovine prostheses demonstrated a pronounced degradation concurrently with ECM deposition and absence of calcification at 6 months postimplantation, in contrast to the rat model [44]. Taken together, these results illustrate the importance of TEVG testing in large animal models and underline major drawbacks of small animal models in this regard.

Regenerated arterial tissue was similar to the contralateral artery, also having three layers resembling intima, media, and adventitia. Importantly, it was fully endothelialised, highly vascularised, and populated by VSMCs and macrophages, two major populations which are, along with endothelial cells, responsible for the arterial homeostasis. The regenerated artery was generally resistant to calcification, but did not contain elastic fibres and hierarchical layers of VSMCs, which contributed to its mechanical incompetence and made it unable to restore tissue integrity upon resorption of the PHBV/PCL scaffold. In contrast, all ePTFE grafts were heavily calcified.

Both endothelial lysate and vascular homogenate of the regenerated artery replacing PHBV/PCL[VEGF-bFGF-SDF]^Hep/Ilo^ grafts showed elevated expression of IL1B, CXCL8, and MMP2 genes in comparison with a contralateral carotid artery, suggestive of a cytokine-driven remodelling, in accordance with previous studies [20,45,46]. Unfortunately, studies measuring interleukin-1b, interleukin-6, or interleukin-8 in TEVGs upon their implantation have not been conducted to date. However, matrix metalloproteinase 2 was found to be upregulated 20 [47,48] and even 80 weeks [47] postimplantation of TEVGs in the pulmonary artery [47] or aorta [48] of lambs. Similar results were obtained after the short-term (≤4 weeks) implantation of TEVGs into the inferior vena cava of mice [49]. Intriguingly, we found a pronounced expression of the genes encoding pro-inflammatory molecules (IL1B, IL6, and ICAM1) as well as the gene for the key transcription factor governing endothelial-to-mesenchymal transition (SNAI2) in the endothelial lysate as compared with vascular homogenate upon normalisation to the expression of the respective genes in the contralateral carotid artery. Yet, neither cytokine release nor endothelial-to-mesenchymal transition have been measured in situ after the implantation of TEVGs. Further research in this direction would require transcriptomic and proteomic profiling (e.g., by means of RNA-seq, dot blotting arrays, or mass spectrometry) of endothelial lysate and vascular homogenate at ascending time points following TEVG implantation.

## 5. Conclusions

The present study demonstrates the suitability of a small-diameter electrospun TEVG fabricated from a biodegradable PHBV/PCL polymer blend, biofunctionalised by the incorporation of bioactive factors (VEGF, bFGF and SDF-1α), and modified with heparin and iloprost to improve antithrombotic properties. Upon implantation into the ovine carotid artery, PHBV/PCL[VEGF-bFGF-SDF]^Hep/Ilo^ grafts exhibited high biocompatibility and calcification resistance along with a moderate haemocompatibility, but were prone to aneurysm formation. Hence, this TEVG prototype needs mechanical reinforcement and further improvement of its antithrombotic coating. Yet, the 50% primary patency at 18 months postimplantation holds promise for the translation of the PHBV/PCL[VEGF-bFGF-SDF]^Hep/Ilo^ vascular graft prototype into future preclinical trials following anti-aneurysmal and antimicrobial modification. Further molecular profiling studies are needed to better understand the arterial regeneration which occurs following the replacement of the PHBV/PCL blend. Relative quantification of endothelial-to-mesenchymal transition and osteogenic transcription factors in comparison with the contralateral ovine carotid artery, as well as interrogation of vascular smooth muscle cell plasticity and macrophage polarisation at ascending time points postimplantation, would be particularly beneficial.

## Figures and Tables

**Table 1:** Sequences of customised primers for RT-qPCR.

Gene	Forward Primer Sequence	Reverse Primer Sequence
IL1B	5′-TGCTGAAGGCTCTCCACCTC-3′	5′-ACCCAAGGCCACAGGAATCTT-3′
IL6	5′-TGTCATGGAGTTGCAGAGCAGT-3′	5′-CCAGCATGTCAGTGTGTGTGG-3′
CXCL8	5′-CTTCCAAGCTGGCTGTTGCTC-3′	5′-ATTTGGGGTGGAAAGGTGTGG-3′
ICAM1	5′-GTCACGGGGAACAGATTGTAGC-3′	5′-TGAGTTCTTCACCCACAGGCT-3′
MMP2	5′-ACCCCGCTACGGTTTTCTCG-3′	5′-ATGAGCCAGGAGCCCGTCTT-3′
KDR	5′-ACAGAACCAAGTTAGCCCCATC-3′	5′-TCGCTGGAGTACACAGTGGTG-3′
NR2F2	5′-GCAAGCGGTTTGGGACCTT-3′	5′-GGACAGGTAGGAGTGGCAGTTG-3′
SNAI2	5′-ACCCTGGTTACTGCAAGGACA-3′	5′-GAGCCCTCAGATTGGACCTG-3′
GAPDH	5′-TGGTGAAGGTCGGAGTGAACG-3′	5′-AGGGGTCATTGATGGCAACG-3′
B2M	5′-CCTTCTGTCCCACGCTGAGT-3′	5′-TGGTGCTGCTTAGAGGTCTCG-3′

**Table 2:** Mechanical properties of PHBV/PCL[VEGF-bFGF-SDF] vascular grafts before and after Hep/Ilo coating in comparison with ePTFE prostheses and IMA.

	PHBV/PCL [VEGF-bFGF-SDF] (*n* = 9) Median (IQR)	PHBV/PCL [VEGF-bFGF-SDF]^Hep/Ilo^ (*n* = 9) Median (IQR)	ePTFE (*n* = 9) Median (IQR)	IMA (*n* = 9) Median (IQR)
Ultimate Tensile Strength, MPa	3.05 (2.90; 3.20) ^&^	3.94 (3.78–3.99) ^&^	22.95 (22.42–23.47) **	2.48 (1.36–3.25) ^&^
Ultimate Tensile Force, N	2.30 (2.20; 2.50) ^#,&^	3.08 (2.94–3.30) ^#,&^	21.10 (20.60–21.60) **	0.92 (0.59–1.72) ^&^
Elongation at Break, %	121.70 (117.1; 129.6) ^#,&^	109,17 (92.29–116.06) ^#,&^	337.00 (332.00–341.80) **	29.72 (23.51–39.62) ^&^
Elastic Modulus, MPa	8.60 (8.00; 9.64) ^#,&^	49.95 (44.90–54.70) *^,#,&^	1.98 (1.36–2.59)	2.42 (1.87–3.19)
Sample Thickness, mm	0.36 (0.34–0.39)	0.39 (0.39–0.40)	0.46 (0.46–0.46)	0.27 (0.24–0.30)

PHBV/PCL—poly(3-hydroxybutyrate-co-3-hydroxyvalerate), PCL—poly(ε-caprolactone), [VEGF-bFGF-SDF]—combination of vascular endothelial growth factor, basic fibroblast growth factor and stromal cell-derived factor 1α, Hep/Ilo—modification with heparin and iloprost, ePTFE—expanded poly(tetrafluoroethylene), IMA—internal mammary artery, IQR—interquartile range. * *p* < 0.05 compared with PHBV/PCL[VEGF-bFGF-SDF] grafts, ^#^ *p* < 0.05 compared with IMA, ^&^ *p* < 0.05 compared with ePTFE prostheses, ** *p* < 0.05 compared with all groups.

**Table 3:** Haemolysis and platelet aggregation measured upon the contact of the blood or platelet-rich plasma with vascular grafts.

Graft Type	Haemolysis, % Median (IQR)	Maximum Platelet Aggregation, % Median (IQR)
Platelet-rich plasma	-	14.61 (13.63–17.72)
PHBV/PCL[VEGF-bFGF-SDF]	0 (0–0)	17.25 (16.30–17.96)
PHBV/PCL[VEGF-bFGF-SDF]^Hep/Ilo^	0.36 (0.36–0.36) **	8.22 (8.13–8.78) *
ePTFE	0.33 (0.21–2.40) **	22.74 (22.45–24.52)

PHBV/PCL—poly(3-hydroxybutyrate-co-3-hydroxyvalerate), PCL—poly(ε-caprolactone), [VEGF-bFGF-SDF]—combination of vascular endothelial growth factor, basic fibroblast growth factor and stromal cell-derived factor 1α, Hep/Ilo—modification with heparin and iloprost, ePTFE—expanded poly(tetrafluoroethylene), IQR—interquartile range. * *p* < 0.05 compared with platelet-rich plasma, PHBV/PCL[VEGF-bFGF-SDF] and ePTFE grafts, ** *p* < 0.05 compared with platelet-rich plasma and PHBV/PCL[VEGF-bFGF-SDF] grafts.

## Data Availability

The data presented in this study are available on request from the corresponding author. The data are not publicly available due to the data form part of an ongoing study.
